# Molecular characteristics and cancer immunity of LRP1B and its relationship with the Hedgehog signaling pathway in colorectal cancer

**DOI:** 10.3389/fimmu.2025.1567102

**Published:** 2025-03-18

**Authors:** Yuan Liu, Yang Zhong, Yaodong Sang, Siqiang Zhu, Kang Xu, Xingyu Zhu, Xiaoling Cui, Xinyu Liu, Xiaohan Wang, Hao Chen, Changqing Jing, Wei Chong, Leping Li

**Affiliations:** ^1^ Department of Gastrointestinal Surgery, Shandong Provincial Hospital Affiliated to Shandong First Medical University, Key Laboratory of Engineering of Shandong Province, Shandong Provincial Hospital, Jinan, China; ^2^ Medical Science and Technology Innovation Center, Shandong First Medical University & Shandong Academy of Medical Sciences, Jinan, China; ^3^ Department of Epidemiology and Health Statistics, School of Public Health, Cheeloo College of Medicine, Shandong University, Jinan, Shandong, China; ^4^ Clinical Research Center of Shandong University, Clinical Epidemiology Unit, Qilu Hospital of Shandong University, Jinan, Shandong, China

**Keywords:** colorectal cancer (CRC), LRP1B, hedgehog (Hh) signaling pathway, immune cell infiltration, tumor microenvironment

## Abstract

**Background:**

Colorectal cancer (CRC) is a malignant tumor of the digestive tract that significantly impacts human health. LDL receptor-related protein 1B (LRP1B) may play a crucial role in tumorigenesis and disease progression.

**Methods:**

We performed a comparative analysis of differential gene expression, mutation patterns, drug sensitivity, and cellular phenotypes across different subgroups with varying LRP1B expression levels. Cellular and molecular experiments were conducted to validate our findings.

**Results:**

Our analysis implicated LRP1B as a tumor suppressor gene. Experimental results confirmed that LRP1B expression was reduced in CRC and its knockdown was associated with poor prognosis. Molecular mechanism studies revealed that LRP1B negatively regulated the Hedgehog (Hh) signaling pathway, influencing cell cycle and apoptosis processes. Single-cell analysis showed significant differences in the infiltration of T cells, B cells, epithelial cells, and myeloid cells between high and low LRP1B expression groups. Immune cell infiltration and drug sensitivity analyses demonstrated that LRP1B plays a crucial role in immunotherapy and targeted therapy, suggesting that restoring LRP1B function could be a promising treatment strategy for CRC.

**Conclusion:**

Our results indicate that LRP1B may function as a tumor suppressor factor in CRC, playing a significant role in mutation, therapy, and immune infiltration. Knockdown of LRP1B activates the Hh pathway in tumor cells, leading to the inhibition of several malignant biological behaviors.

## 1 Introduction

In 2020, among 36 cancers across 185 countries, colorectal cancer (CRC) ranked third in incidence and second in mortality (1). Despite growing interest in therapeutic targets for chemotherapy and immunotherapy (2), including the gut microbiota (3) and immune checkpoint inhibitors (4–6), clinical benefits remain limited. LDL receptor-related protein 1B (LRP1B) has been identified as a negative prognostic factor in various cancers, with its mutation status and expression levels playing a crucial role (7–9). LRP1B has been validated as a key component of a mutational prognostic signature, potentially serving as an independent predictor of recurrence and prognosis in patients with stage III colon cancer (10, 11). However, its specific mechanism remains unclear.

A genome-wide significance (GWS) meta-analysis for CRC involving over 125,000 individuals identified the Hedgehog (Hh) signaling pathway as a key genetic component of CRC and highlighted its role in immune function (12). The Hh signaling pathway regulates numerous tissue patterning events during developmental processes through interaction among secreted Hh ligands, the transmembrane receptor protein patched (PTCH), the transmembrane protein smoothened (SMO), the suppressor of fused (SUFU), and Gli transcription factors, including GLI1–3 in vertebrates (13). Three mammalian counterparts of the Hh have been identified: Sonic hedgehog (SHH), Indian hedgehog (IHH), and Desert hedgehog (DHH), with SHH serving as the primary organizing center (14). When Hh ligands bind to PTCH, primarily PTCH1, the ligand–receptor complexes are internalized and degraded, leading to SMO phosphorylation. This process facilitates SUFU-GLI dissociation, releasing GLI and inducing target gene transcription (15). The pathway regulates a wide array of genes involved in development, cell cycle control, and apoptosis, either directly or indirectly (16).

The immune microenvironment plays a crucial role in the proliferation and metastasis of tumors (17, 18). With the clinical success of cancer immune checkpoint blockade (ICB), infiltrating immune cells, particularly T cells, have gained increasing attention (19, 20). Moreover, novel multidimensional analysis platforms, such as single-cell RNA sequencing and high-dimensional flow cytometry, have facilitated the study of tumor-infiltrating immune cell heterogeneity (21).

Our bioinformatics analysis revealed that LRP1B is negatively associated with the prognosis of CRC patients. Additionally, LRP1B may regulate the malignant behavior of cancer cells through the Hh signaling pathway. We also examined the relationship between the LRP1B expression and immune cell infiltration using single-cell expression analysis. These findings suggest that LRP1B could serve as a marker for assessing T-cell infiltration and predicting prognosis and immunotherapy efficacy in CRC patients.

## 2 Materials and methods

### 2.1 Data collection and preprocessing

Gene expression data and clinical features of CRC samples were retrieved from public datasets of the NCBI GEO (https://www.ncbi.nlm.nih.gov/geo/) and The Cancer Genome Atlas (TCGA) (https://cancergenome.nih.gov/) databases. The expression data of the GSE39582 dataset, obtained from the GEO database on the Affymetrix GPL570 platform, underwent preprocessing using the RMA algorithm with the “affy” package. Duplicate probes were merged by calculating the median value, thereby reducing redundancy in the expression data. As for the TCGA datasets, the data of TCGA-Colon Adenocarcinoma (COAD) and TCGA-Rectal Adenocarcinoma (READ) datasets, which together form the TCGA-CRC cohort, were downloaded from cBioPortal (https://www.cbioportal.org/) and the USCS Xena (https://xenabrowser.net/datapages/) database. The transcriptome data have been processed and quantified into FPKM format to estimate transcript abundances and provide gene-level expression estimates for downstream analysis. To enhance the precision of detecting the impact of LRP1B expression levels, we applied a filtering process to include only samples with expression levels above 0. The samples were then categorized into high- and low-expression groups based on the median expression level.

### 2.2 Single-cell expression analysis

We collected the bulk RNA-seq and single-cell RNA-seq data from a CRC study by Khaliq et al. (22), which dissected the tumor microenvironment with LRP1B dysregulation. Samples with matched bulk and single-cell RNA-seq were retained, and a total of 22,344 high-quality CRC cells from 15 samples were subjected to further analysis using the Seurat package. We applied the t-Distributed Stochastic Neighbor Embedding (tSNE) method to visualize the high-dimensional data. The major cell type and subtype annotation were adopted from the study by Ateeq et al. Additionally, we employed the Uniform Manifold Approximation and Projection (UMAP) method to visualize the data from the Single Cell Expression Atlas (SCEA, https://www.ebi.ac.uk/gxa/sc/home) database, which was derived from a study by Lee et al. (23). Both tSNE and UMAP are nonlinear dimensionality reduction techniques that map high-dimensional data to a lower-dimensional space. These approaches allow for the visualization of datasets and the reduction of data dimensionality, facilitating further analysis (24, 25). The single-cell analysis plots using the tSNE method were customized based on the cell types labeled by Khaliq et al., while other plots using the UMAP method followed the cell types labeled by Lee et al.

### 2.3 Gene set enrichment analysis

The “limma” package was used to evaluate the differential expression of more than 20,000 genes in samples from different expression groups. The gene expression data were processed using the lmFit and eBayes functions to calculate differential statistics with the package (26). The ranked logFC values produced by limma were used to perform gene set enrichment analysis (GSEA) with a fast GSEA algorithm against Hallmark Gene Sets and Kyoto Encyclopedia of Genes and Genomes (KEGG) gene sets (27).

### 2.4 PPI network associated with different expression and prognosis

We determined differentially expressed genes (DEGs) between high- and low-expression groups using the limma package (26). The expression-related DEGs were further filtered by intersecting them with DEGs between tumor and nontumor tissues. The normalized gene expression data were processed using the lmFit and eBayes functions to calculate expression statistics. The significance criteria for DEGs were set as a false discovery rate (FDR) of less than 0.001 and a log2-fold change of more than 0.5 and 2, respectively.

The Search Tool for the Retrieval of Interacting Genes/Proteins (STRING, https://string-db.org/) database was used to investigate gene interactions and visualization (28, 29). Prognostic DEGs obtained above were submitted to the STRING database to analyze their protein–protein interactions (PPI). The network was imported into Cytocape software to organize the interactions and exclude nodes without betweenness.

### 2.5 Immune cell infiltration and signature estimation

The deconvolution approach xCell algorithm was selected to characterize molecular features related to immunology between expression groups (30). xCell utilizes a gene expression signature matrix that includes marker genes specific to various cell types. Its output provides estimates of the abundance of different immune and stromal cell types in each sample, which can be further analyzed and integrated with other clinical or molecular data.

“The IOBR” package integrates 255 published signature gene sets related to the tumor microenvironment, tumor metabolism, m6A, exosomes, microsatellite instability, and tertiary lymphoid structures (31). The PCA method was used in the feature score evaluation process in our study.

### 2.6 Estimation and validation of drug sensitivities

The “oncoPredict” package was used to build the drug sensitivity prediction procedure. Imputations were performed based on the expression matrix of a training set with known drug treatment information from the Genomics of Drug Sensitivity in Cancer (GDSC) database (32). The drug sensitivity scores of the samples were calculated using Ridge regression.

### 2.7 Genomic operation on the mutational signature

TCGA-CRC genomic data were curated using the “TCGAbiolink” package. The “maftools” package was used for mutational landscape depiction and signature extraction (33). The extract signatures function, based on Bayesian variant nonnegative matrix factorization, factorized the mutation portrait matrix into two nonnegative matrices: “signatures” and “contributions”, where signatures represent mutational processes and contributions represent the corresponding mutational activities (34). The signature enrichment function can automatically determine the optimal number of extracted mutational signatures and assign them to each sample based on mutational activities. The extracted mutational portrait of CRC was compared and annotated using cosine similarity analysis against the Catalogue of Somatic Mutations in Cancer (COSMIC) database (35).

### 2.8 Cell culture and transfection

The LS180 cell line was obtained from the Cell Resource Center, Peking Union Medical College (which is part of the National Science and Technology Infrastructure, National Biomedical Cell-Line Resource, NSTI-BMCR. http://cellresource.cn). It was grown in MEM medium (KeyGEN BioTECH, Jiangning District, Nanjing City, Jiangsu Province, China) containing 10% fetal bovine serum (PAN), penicillin (100 U/mL, Thermo Fisher, Meiyou Road, China (Shanghai) Pilot Free Trade Zone), and streptomycin (100 U/mL, Thermo Fisher). HCT116 cell line (Procell, Wuhan City, Hubei Province, China) was grown in McCoy’s 5A medium (KeyGEN BioTECH) supplemented with 10% fetal bovine serum (PAN), penicillin (100 U/mL, Thermo Fisher), and streptomycin (100 U/mL, Thermo Fisher). The HCT116 cell line was obtained on 13 May 2023 and tested by STR. Other colorectal cell lines (RKO, HCT15, SW480, SW620, DLD-1) were obtained from ATCC. The RKO cell line was grown in RPMI-1640 medium (Gibco), while the other cell lines were cultured in DMEM (Gibco), with 10% fetal bovine serum (PAN), penicillin (100 U/mL, Thermo Fisher), streptomycin (100 U/mL, Thermo Fisher). All cells were cultured in 95% air and 5% CO_2_ at 37°C.

The LRP1B-RNAi and negative control lentiviruses were purchased from Shanghai Jikai Company (Shanghai, China). The cells were infected with lentivirus for 24 h and selected with puromycin (1.5 mg/mL, MedChemExpress, Zhangheng Road, Pudong New District, Shanghai, China) for 7 days.

### 2.9 Real-time quantitative PCR

Total RNA from cells was isolated using a Trizol reagent (Vazyme, Kechuang Road, Nanjing Economic and Technological Development Zone, China). RNA was reversely transcribed into cDNA using HiScript III RT SuperMix for qPCR (Vazyme, China), following the manufacturer’s instructions. Quantitative real-time polymerase chain reaction (qRT-PCR) was performed using Applied Biosystems QuantStudio 1 Real-Time PCR system (Applied Biosystems, Thermo Fisher) with ChamQ Universal SYBR qPCR Master Mix (Vazyme, China). The relative expression levels of mRNA were calculated by using the 2^−ΔΔCt^ method, where a higher 2^−ΔΔCt^ indicates higher expression.

### 2.10 Western blotting

Cells were lysed with RIPA buffer (Solarbio, Beijing, China) containing 1% PMSF and 1% Phosphatase Inhibitor Cocktail I. After centrifugation, total protein was quantified using a BCA protein assay kit (Solarbio). Equivalent amounts of protein were separated by SDS-PAGE and transfected to PVDF membranes. The membranes were incubated with primary antibodies at 4°C overnight, followed by incubation with the corresponding secondary antibodies at room temperature for 1 h the next day. Immunoreactive bands were visualized using an ECL detection kit (Vazyme, China) and imaged with the Amersham ImageQuant 800 system.

The following primary antibodies were purchased: SHH (1:1,000, 2207T, Cell Signaling Technology, Shengxia Road, Pudong New Area, Shanghai, China), PTCH1 (1:1,000, 2468T, Cell Signaling Technology, USA), PTCH2 (1:1,000, 2470T, Cell Signaling Technology, USA), SMO (1:1,000, 92981T, Cell Signaling Technology, USA), SUFU (1:1,000, 2520T, Cell Signaling Technology, USA), GLI1 (1:1,000, 3538T, Cell Signaling Technology, USA), CHEK2 (1:1,000, 13954-1-AP, Proteintech, Chicago, USA), E2F1 (1:200, sc-251, Santa Cruz Biotechnology, USA), Cyclin D1 (1:1,000, 2978S, Cell Signaling Technology, USA, Jianye Road Pudong New District, Shanghai, China), Cyclin B1 (1:1,000, 12231S, Cell Signaling Technology, USA), p53 (1:1,000, 2527S, Cell Signaling Technology, USA), CDK1 (1:2,000, 19532-1-AP, Proteintech, Chicago, USA), CDK2 (1:1,000, 2546S, Cell Signaling Technology, USA), p21 (1:1000, 2947S, Cell Signaling Technology, USA), BCL-2 (1:1,000, 15071S, Cell Signaling Technology, USA), and β-actin (1:2,000, 20536-1-AP, Proteintech, Chicago, USA).

### 2.11 Cell Counting Kit-8 assays

To assess cell viability, 10 µL/well of Cell Counting Kit-8 (CCK8) reagent (DojinDo, Japan) was added to a 96-well plate containing 3,000 cells per well, followed by incubation at 37°C for 2 h. The absorbance value of each pore was measured at 450 nm using a Multiskan Sky microplate reader (Thermo Scientific) at 0, 24, 48, 72, and 96 h. Cell viability was calculated using GraphPad Prism 8 software. All experiments were repeated three times.

### 2.12 Colony formation assays

Based on the growth rate of the cells, different seeding densities were selected for the six-well plates, with 3,000 cells per well for LS180 and 2,000 cells per well for HCT116. The culture medium was refreshed every 3 days. After 7 days of culture, the plates were washed twice with 4°C precooled PBS, fixed with 4% paraformaldehyde at 4°C for 30 min, and stained with crystal violet for 30 min. After drying, the colonies were photographed, and their numbers were quantified using Image J software. Each experiment was conducted in triplicate.

### 2.13 Apoptosis and cell cycle analysis

Apoptosis and cell cycle status were evaluated using the BD Pharmingen™ PE Annexin V Apoptosis Detection Kit I (BD Biosciences, Nanjing West Road, Shanghai, China) and the KeyGEN BioTECH Cell Cycle Detection Kit, respectively. For apoptosis assessment, cells were resuspended in 200 µL of binding buffer and incubated with 10 µL of 7-AAD and 10 µL of PE Annexin V for 15 min at room temperature in the dark. For cell cycle analysis, cells were incubated with 500 µL of PI/RNase A for 30 min. Samples were then analyzed using a CytoFLEX S Flow Cytometer (Beckman Coulter, Eshan Road, China (Shanghai) Pilot Free Trade Zone).

### 2.14 Statistical analysis

Data processing, statistical analysis, and plotting were conducted using R 4.2.3. The Wilcoxon rank-sum test or *t*-test was used to compare differences between two groups for quantitative data, while two-sided Fisher’s exact tests were applied to analyze categorical variables. Kaplan–Meier analysis and Cox regression analysis were performed using the “survival” and “survminer” packages. All statistical tests were two-sided, with *p* < 0.05 considered statistically significant. Error bars represent 95% confidence intervals. The Benjamini–Hochberg method was used to control the FDR for multiple hypothesis testing where appropriate. Experiment statistical analyses were performed using GraphPad Prism 8 software, with each experiment repeated at least three times. Statistical significance was assessed using the Student’s *t*-test or Wilcoxon’s rank-sum test, with *p* < 0.05 considered statistically significant.

## 3 Results

### 3.1 The implications of LRP1B expression in CRC

**Figure 1:**
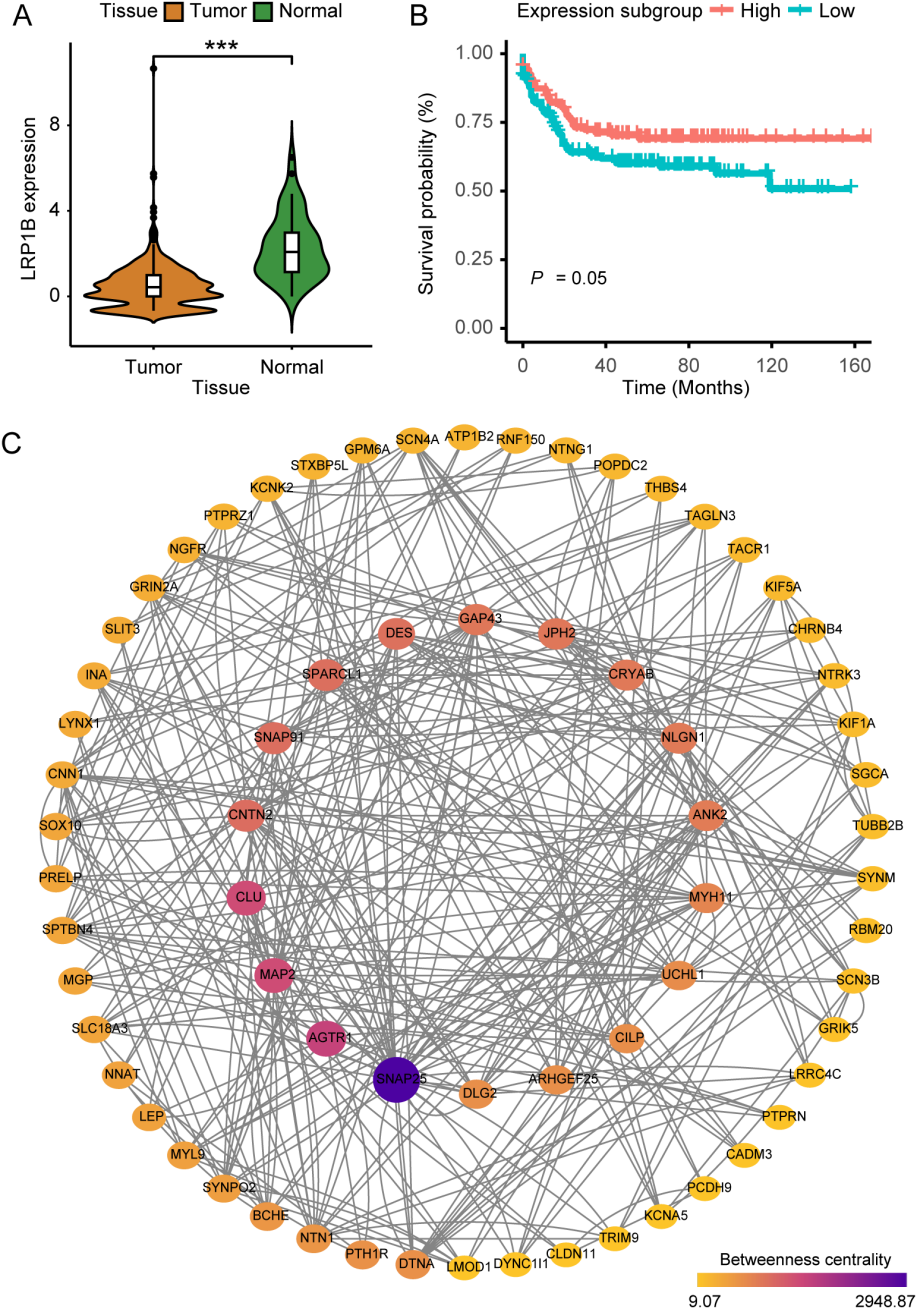
The implications of LRP1B in colorectal cancer. **(A)** Violin plot showing the distribution of LRP1B expression between tumor tissues and adjacent normal tissues. **(B)** Kaplan–Meier curves depicting progression-free survival (PFS) across expression subgroups. **(C)** Protein–protein interaction (PPI) network was constructed with 68 filtered DEGs, where node size and color indicate betweenness. ^***^ *p* < 0.001.

To explore the dysregulation of LRP1B, we analyzed CRC sample data and observed significant differences in expression levels between cancer tissues and cancer-adjacent normal tissues from public datasets of GEO and TCGA. Specifically, the expression level of LRP1B was significantly lower in cancer tissues compared to several paired normal tissues (**Figure 1A**), indicating a potential role for LRP1B in inhibiting tumor development. Furthermore, single-cell sequencing analysis revealed that LRP1B expression was predominantly low in tumor immune cells, falling below the expression cutoff (**Supplementary Figure S1A**). When compared to adjacent normal tissues as a reference, few stromal, glial, and endothelial cells exhibited detectable LRP1B expression (**Supplementary Figure S1B**). Subsequently, the significant downregulation of LRP1B was verified using the HPA platform (**Supplementary Figure S1C**). These findings support the conclusion that LRP1B is primarily expressed in normal tissues. Although survival analyses grouped by median expression did not show significant results (**Supplementary Figure S1D**), possibly due to a small sample size, further analysis—including only samples with expression above the upper quartile and below the lower quartile—revealed statistically significant findings. This suggests that the downregulation or loss of LRP1B expression is associated with poor prognosis and decreased patient survival in CRC (**Figure 1B**).

We identified a total of 1,590 DEGs between tumor and normal samples (FDR < 0.001, log2-fold change > 2). Additionally, 931 DEGs were found to be related to LRP1B expression (FDR < 0.001, log2-fold change > 0.5). We subsequently overlapped the two sets of DEGs, culminating in a consolidated list of 436 genes. After performing Cox regression analysis (153 genes left, **Supplementary Table S1**) and removing isolated nodes and genes with zero betweenness, 68 genes were identified and utilized to form the PPI network (**Supplementary Table S2**). Genes such as KIF5A, SOX10, MYL9, TRIM9, MYH11, and SNAP25 are known to play important roles in biological development, suggesting that LRP1B may also be a key factor in cancer (**Figure 1C**).

### 3.2 Relationship between LRP1B expression with mutation landscapes in CRC

**Figure 2:**
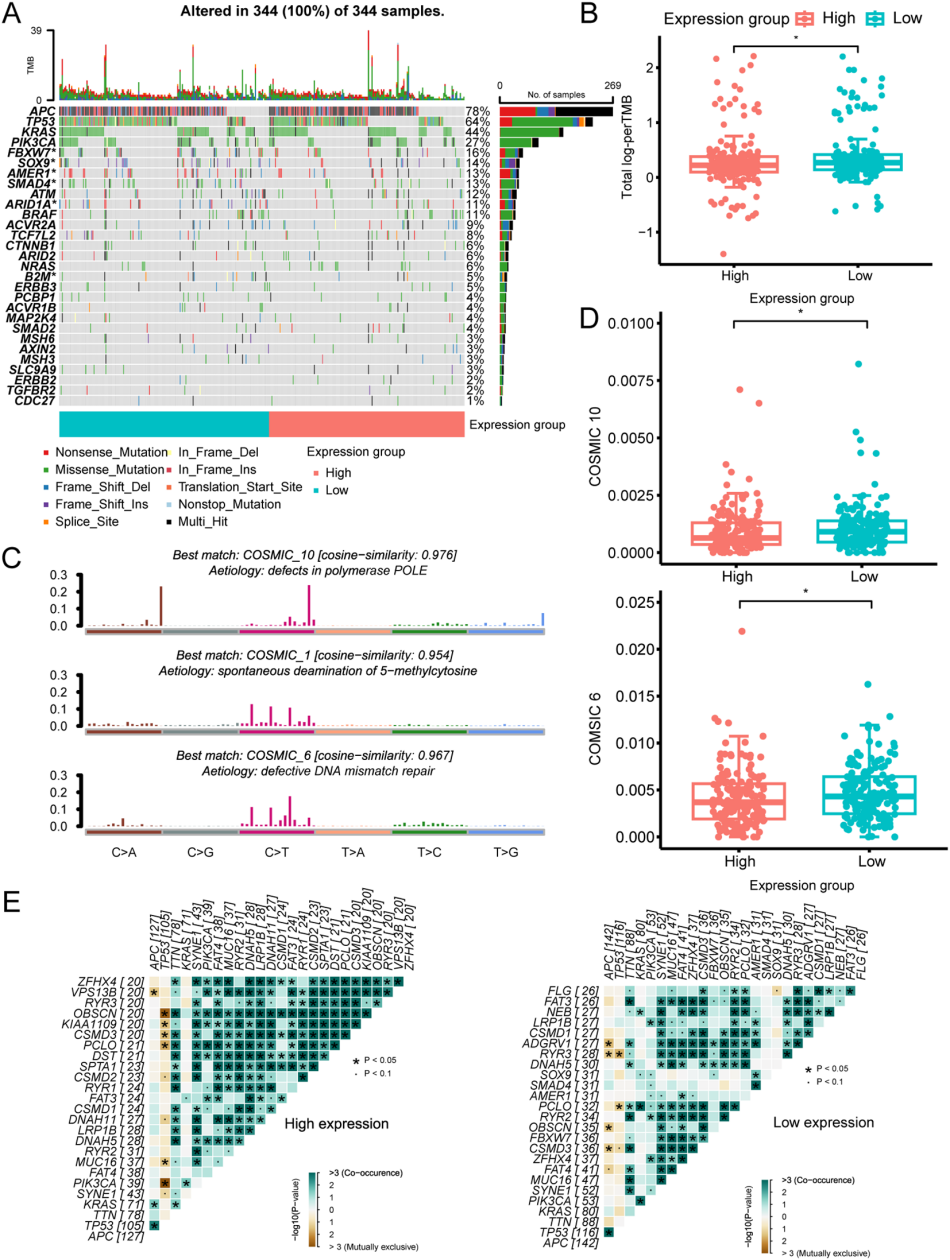
The mutational landscape across different LRP1B expression groups. **(A)** Oncoplot illustrating the distribution of somatic mutations (SNV/indel) and copy number variation (CNV) events in frequently mutated genes. **(B)** Tumor mutation burden distribution across different groups. **(C)** Annotations of curated mutational signatures. **(D)** Distribution of SBS10 and SBS6 mutational signatures across different groups. **(E)** Somatic interactions of the top 25 mutated genes in high- and low-expression groups. ^*^ *p* < 0.05.

The mutational landscape analysis included mutational frequencies and tumor mutation burden (TMB) levels (**Supplementary Figure S2A**). We found that six genes (B2M, ARID1A, SMAD4, AMER1, SOX9, and FBXW7) had higher mutation frequencies in the low-expression group among the commonly mutated genes in CRC (**Figure 2A**). Furthermore, the low-expression group also had a high TMB level (**Figure 2B**). To explore the underlying mutational processes, we curated mutation processes against the COSMIC database using somatic genomic alteration data and found three signatures—COSMIC 10, 1, and 6—were best enriched based on NMF analysis cophenetic metric and cosine similarity (**Supplementary Figures S2B, C**). The three signatures were separately annotated as defects in polymerase POLE, spontaneous deamination of 5-methylcytosine, and defective DNA mismatch repair (**Figure 2C**). Importantly, COSMIC 10 and 6 scored highly in the low-expression group compared to the high-expression group (**Figure 2D**). Additionally, while the low-expression group exhibited a higher TMB than the high-expression group, it is noteworthy that the high-expression group had a higher frequency of co-mutations. This suggests a greater likelihood of multiple genes being simultaneously affected in the high-expression group (**Figure 2E**). Overall, these findings indicate that LRP1B expression levels may influence the occurrence and pattern of mutations.

### 3.3 The significance of LRP1B in drug sensitivity and immune infiltration

**Figure 3:**
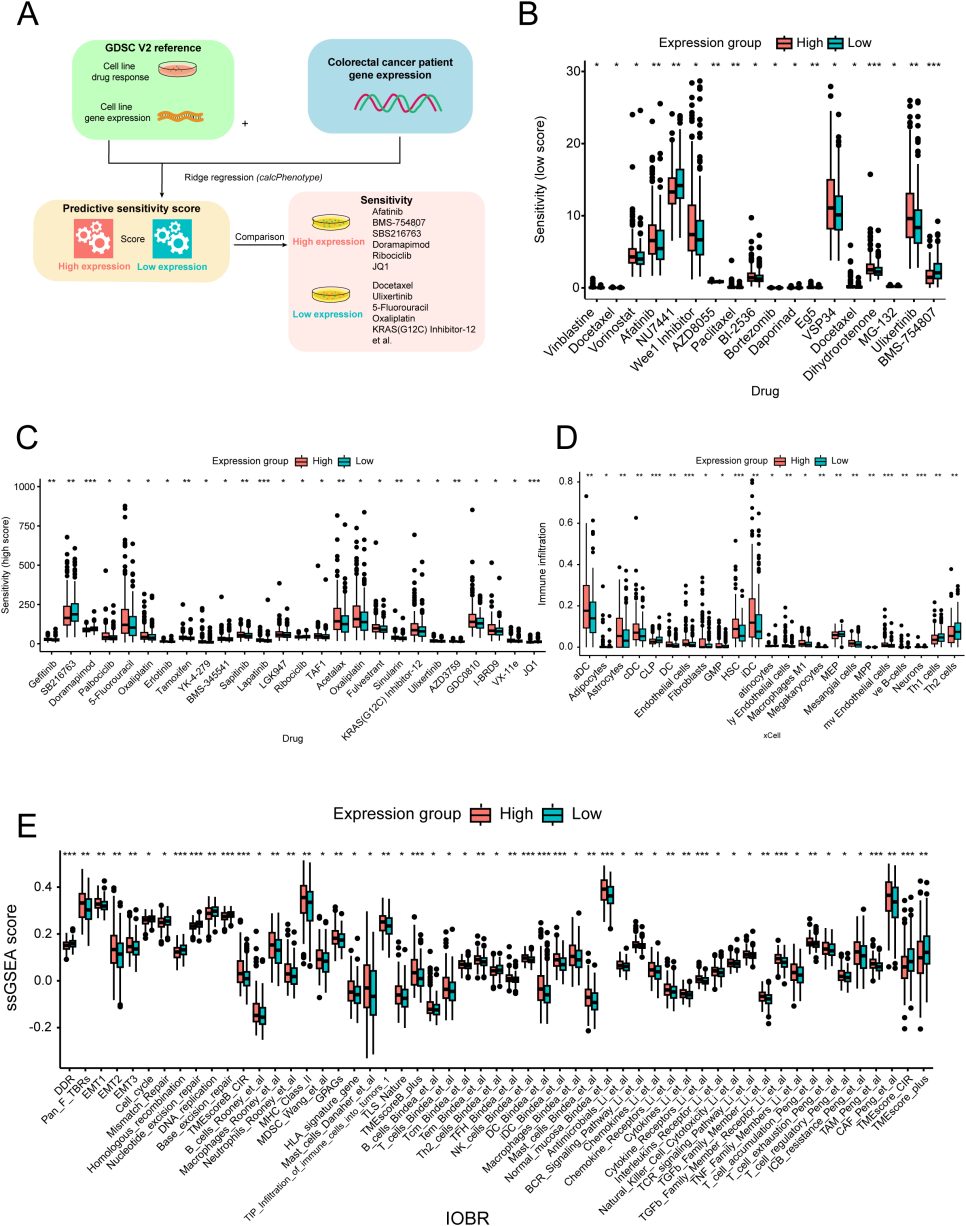
Drug sensitivity and immune infiltration analysis across different expression groups. **(A)** Workflow of the drug sensitivity prediction procedure. Distribution of the relatively low- **(B)** and high-estimated **(C)** drug sensitivity scores across different expression groups. **(D)** Immune cell infiltration score distribution across different expression groups. **(E)** Fraction of oncology-immune signatures across expression groups based on the ssGSEA algorithm. ^*^ *p* < 0.05; ^**^ *p* < 0.01; ^***^ *p* < 0.001.

The drug sensitivity scores obtained from the GDSC database were used to assess the variation in the distribution of individual drugs across different expression groups (**Figure 3A**). Out of the 198 drugs analyzed, 44 exhibited significant differences between the expression groups. Several drugs used for the treatment of CRC (such as 5-fluorouracil, acetalax, and oxaliplatin) had high sensitivity scores in the high-expression group, whereas doramapimod and ribociclib showed the opposite trend (**Figures 3B, C**).

Furthermore, we utilized the xCell method to analyze the immune infiltration landscape across different expression groups. Interestingly, we observed a higher infiltration of dendritic cells, endothelial cells, and macrophages in the high-expression group, whereas helper T cells (Th1 and Th2) were more abundant in the low-expression group (**Figure 3D**). Additionally, comprehensive oncology-immune signature analysis (IOBR) revealed an overall accumulation of immune cells in the high-expression group, except for helper T cells (**Figure 3E**). Overall, these findings suggest that LRP1B expression may influence immune infiltration patterns and related signature characteristics, with potential implications for immune response modulation.

### 3.4 The expression of LRP1B was associated with different cellular phenotypes in tumor microenvironment.

**Figure 4:**
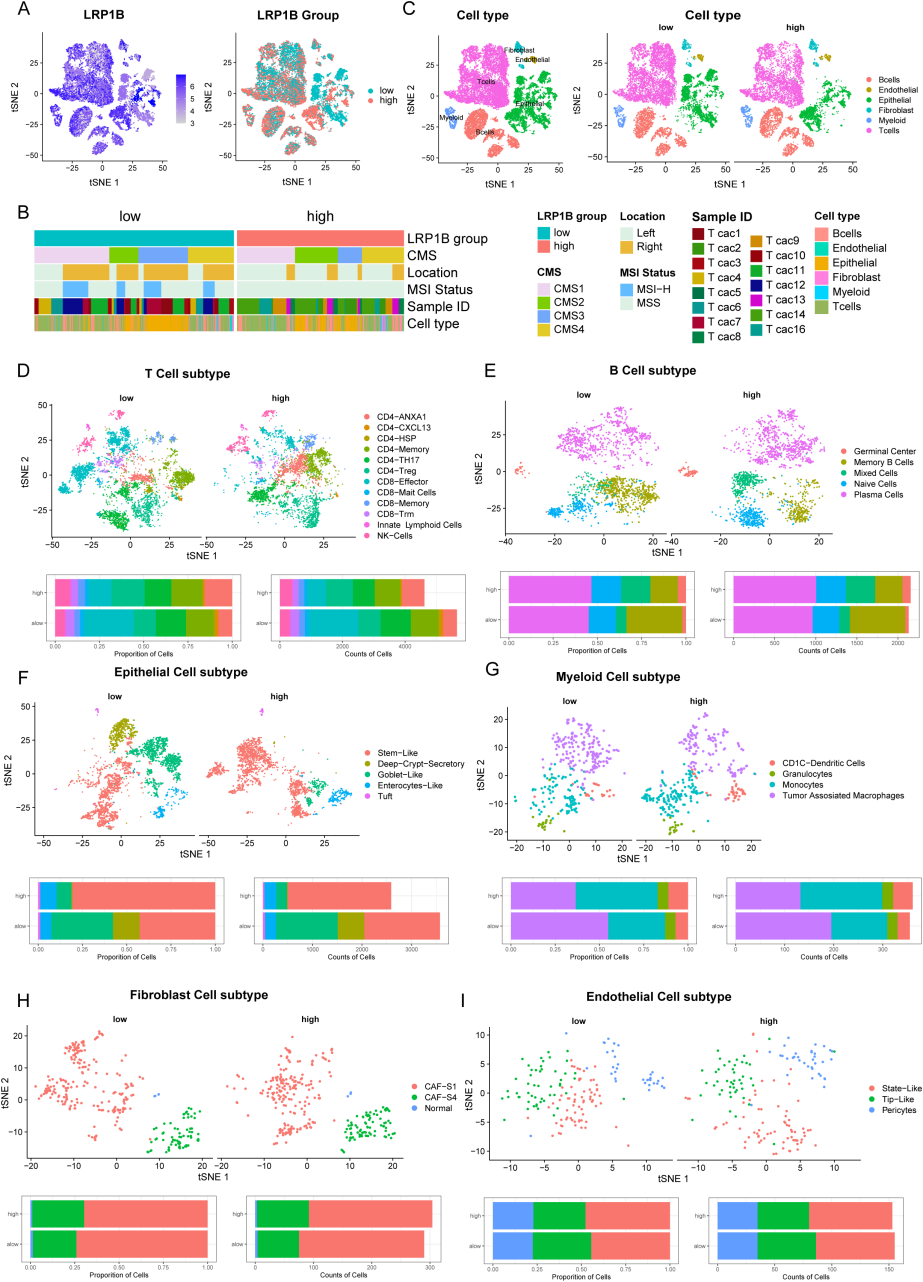
Single-cell analysis of LRP1B expression and immune cell infiltration. **(A)** tSNE plots showing LRP1B expression in 22,344 single cells, categorized into high- and low-expression groups based on bulk RNA-seq. **(B)** Cells clustered by patient, consensus molecular subtypes (CMS), location, and MSI status. **(C)** Cells were clustered into six major cell types, differentially distributed in high and low LRP1B expression groups. Cell subpopulations (upper) and bar plots of cell proportion and counts (lower) for T cells **(D)**, B cells **(E)**, epithelial cells **(F)**, myeloid cells **(G)**, fibroblast cells **(H)**, and endothelial cells **(I)**.

To further understand the association between LRP1B dysregulation and tumor immune microenvironment alteration, we performed a combined bulk RNA-seq and single-cell RNA-seq analysis based on 15 racially diverse, treatment-naïve CRC patient tissue samples (22). A total of 22,344 high-quality single cells were analyzed to profile the immune cell composition across different LRP1B expression statuses. We divided the CRC cells into high and low subgroups according to the expression level of LRP1B in bulk RNA-seq, which matched the cellular distribution in scRNA-seq (**Figure 4A**). Next, we reorganized the CRC cells based on LR1PB expression, consensus molecular subtypes (CMS), tumor location, and MSI status (**Figure 4B**). The CMS comprises four types: CMS1 (microsatellite instability immune, 14%), characterized by hypermutation, microsatellite instability, and strong immune activation; CMS2 (canonical, 37%), epithelial, with marked WNT and MYC signaling activation; CMS3 (metabolic, 13%), epithelial, with evident metabolic dysregulation; and CMS4 (mesenchymal, 23%), featuring prominent transforming growth factor-β activation, stromal invasion, and angiogenesis (36). We discovered that CMS2 tended to cluster in the high-expression group, while CMS4 was more frequent in the low-expression group (**Supplementary Figure S3A**). Regarding cell cycle phase analysis, cells in the G1 stage were more enriched in the high-expression group (**Supplementary Figure S3B**). Furthermore, we compared the distribution of the six major cell types, as annotated by Khaliq et al. (22), between the LRP1B high- and low-expression subgroups. We found that T cells, B cells, epithelial cells, and myeloid cells were significantly differentially distributed across the LRP1B subgroups (**Figure 4C**). Therefore, we further analyzed the counts and proportions of cell subpopulations, subdividing them into T cells, B cells, epithelial cells, myeloid cells, endothelial cells, and fibroblast cells, respectively (**Supplementary Figures S3D, E**). Comparative analyses showed that the cell subcluster distributions of CD4+ T cells, CD8+ T cells (**Figure 4D**), memory B cells (**Figure 4E**), stem-like epithelial cells (**Figure 4F**), and tumor-associated macrophages (**Figure 4G**) were significantly increased in the LRP1B high-expression group. However, the relationship between endothelial cells and fibroblast cells was inconspicuous (**Figures 4H, I**).

### 3.5 LRP1B regulates the cell proliferation, apoptosis, and cell cycle in colorectal cancer cells

**Figure 5:**
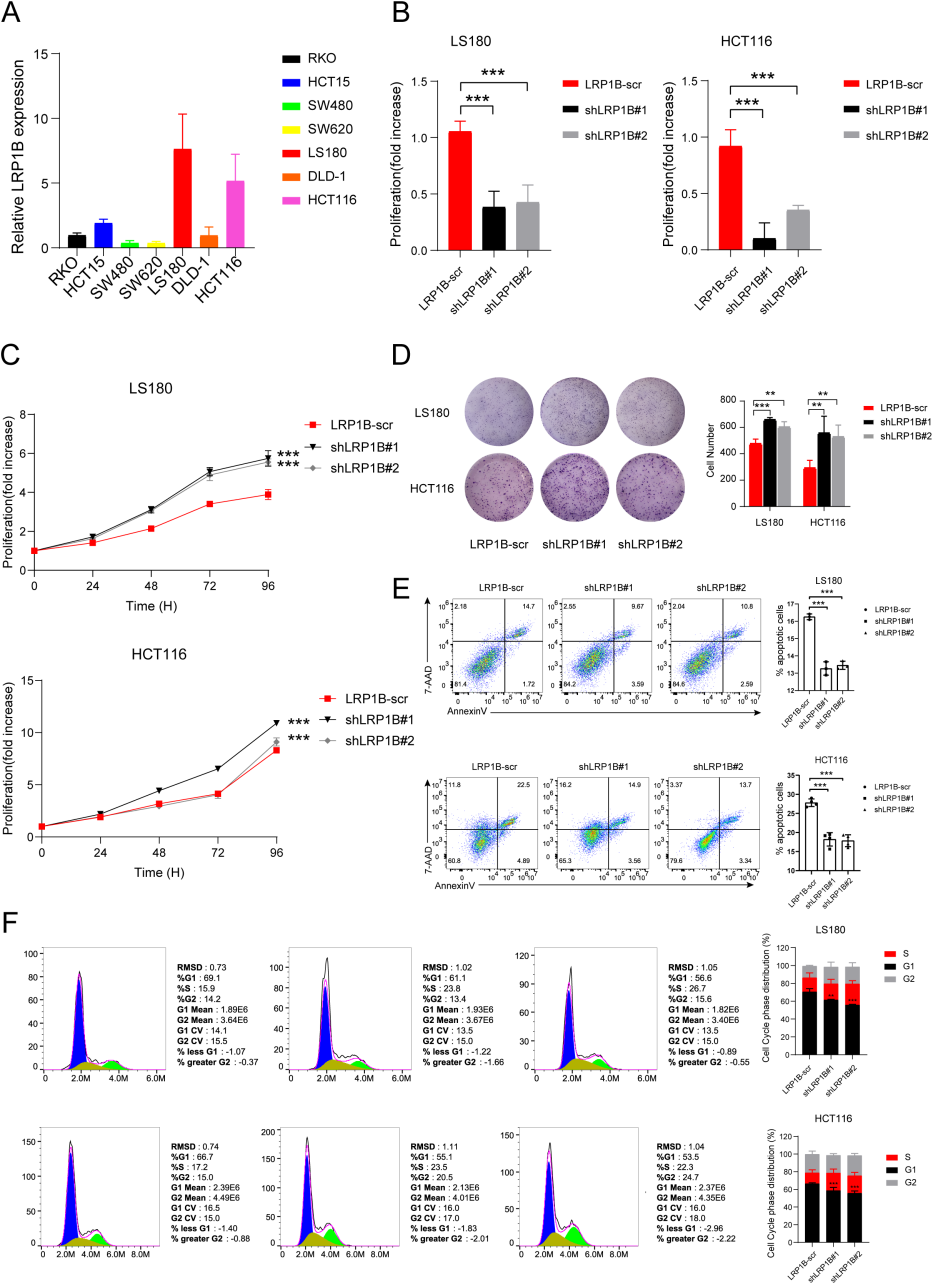
The function of LRP1B in CRC cell lines. **(A)** LRP1B expression levels in different CRC cell lines using quantitative-PCR (qPCR) analysis. **(B)** Confirmation of LRP1B knockdown in LS180 and HCT116 cells using q-PCR analysis. Cell proliferation assay in LS180 and HCT116 cells transfected with LRP1B-scr (negative control), shLRP1B#1, and shLRP1B#2 (LRP1B knockdown), assessed by the cell counting kit-8 (CCK8) assay **(C)** and colony formation **(D)**. Flow cytometric analysis in LS180 and HCT116 cells transfected with LRP1B-scr, shLRP1B#1, and shLRP1B#2 through cell apoptosis **(E)** and cell cycle experiments **(F)**. ^**^ *p* < 0.01; ^***^ *p* < 0.001.

Firstly, we extracted mRNA from different colorectal cancer cell lines (RKO, HCT15, SW480, SW620, LS180, DLD-1, and HCT116) and tested the expression of LRP1B. The qPCR results showed that the expression level of LRP1B in the LS180 and HCT116 cell lines was significantly higher than in the other cell lines (**Figure 5A**). We transfected LS180 and HCT116 cell lines with LRP1B-RNAi and negative control lentiviruses and used the qPCR analysis to verify the transfection efficiency (**Figure 5B**). We then performed CCK8 and colony formation assays to assess the effect of LRP1B on cell proliferation. The proliferation rate was significantly higher in the shLRP1B group than in the control group (**Figure 5C**), and the colony numbers in the shLRP1B group were greater than those in the control group (**Figure 5D**), indicating that knocking down LRP1B can increase cell proliferation. Flow cytometric analysis showed that knocking down LRP1B led to a decrease in apoptotic cells (**Figure 5E**), a decrease in the proportion of cells in the G0/G1 phase, and an increase in the S phase, suggesting that the cell cycle was accelerated in shLRP1B group (**Figure 5F**).

### 3.6 LRP1B inhibits the Hedgehog pathway in colorectal cancer cells

**Figure 6:**
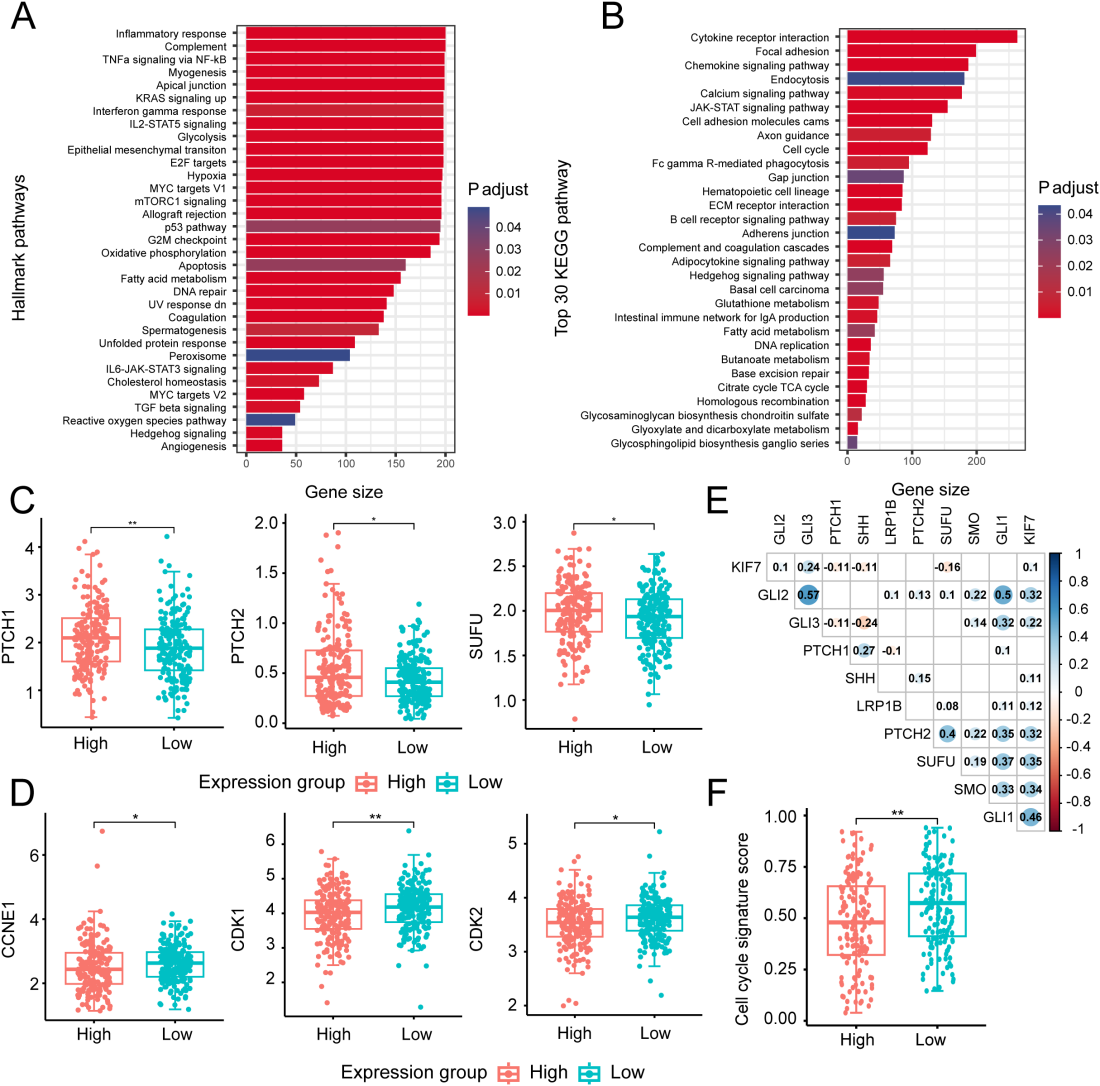
Biological processes and key molecular analysis of the Hedgehog signaling and cell cycle-related pathways. Bar plot presenting the results of HALLMARK **(A)** and top 30 KEGG **(B)** biological pathway enrichment analyses across different expression groups. Distribution of several key gene expressions associated with Hedgehog signaling **(C)** and cell cycle process **(D)** in different groups. **(E)** Correlation matrix illustrating relationships among key molecules in the Hedgehog signaling pathway and the cell cycle process. The size and color intensity of the circles represent the strength of correlation, with numbers inside the circles indicating the correlation coefficients (only statistically significant coefficients are displayed). **(F)** Distribution of cell cycle signature scores across different expression groups. ^*^ *p* < 0.05; ^**^ *p* < 0.01.

To investigate the potential mechanism underlying LRP1B expression, we performed pathway enrichment analysis using HALLMARK and KEGG gene sets and identified several significantly different biological pathways between the high and low LRP1B groups. The results showed that the Hedgehog signaling pathway and cell cycle-related pathways were enriched in the HALLMARK database, including E2F targets, the p53 pathway, and the G2M checkpoint (**Figure 6A**). The KEGG dataset analysis consistently revealed the enrichment of the Hedgehog signaling pathway and cell cycle pathway across different gene sets (**Figure 6B**). Together, we also found that cell cycle- and DNA repair-related processes (such as DNA damage response, homologous recombination, and DNA replication) exhibited a high score in the low LRP1B expression group, while the epithelial–mesenchymal transition process was enriched in the high LRP1B expression group (**Figure 3E**). Notably, the Hedgehog signaling pathway was enriched in both the HALLMARK and KEGG gene sets. Similarly, there is considerable evidence of interaction between the Hh signaling pathway and several other critical signaling cascades across various tumor types (37), such as the Notch pathway (38, 39), the Wnt pathway (40, 41), the RAS signaling pathway (42, 43), epithelial–mesenchymal transition (EMT) (44). Subsequently, we hypothesized that LRP1B exerts its influence by modulating the Hh signaling pathway. To further investigate the association between this pathway and LRP1B, we examined the expression levels of key molecules, including PTCH1, PTCH2, and SUFU, and found that they were significantly upregulated in the high LRP1B expression group (**Figure 6C**). Furthermore, we examined the expression levels of crucial cell cycle genes across the different expression groups. Significantly, CCNE1, CDK1, and CDK2 exhibited higher expression levels in the low LRP1B expression group (**Figure 6D**). Correlation analysis also showed that LRP1B was positively correlated with several Hedgehog signaling pathway molecules, such as PTCH2, SUFU, SMO, and GLI1 (**Figure 6E**). We applied GSE39582 cohort analysis and found that the cell cycle pathway was also enriched (**Supplementary Figure S4**). In addition, we downloaded the cell cycle signature score data from Teresa et al. Their study found that the cell cycle signature score was significantly higher in the low LRP1B expression group (**Figure 6F**), which further confirms our findings (45).

**Figure 7:**
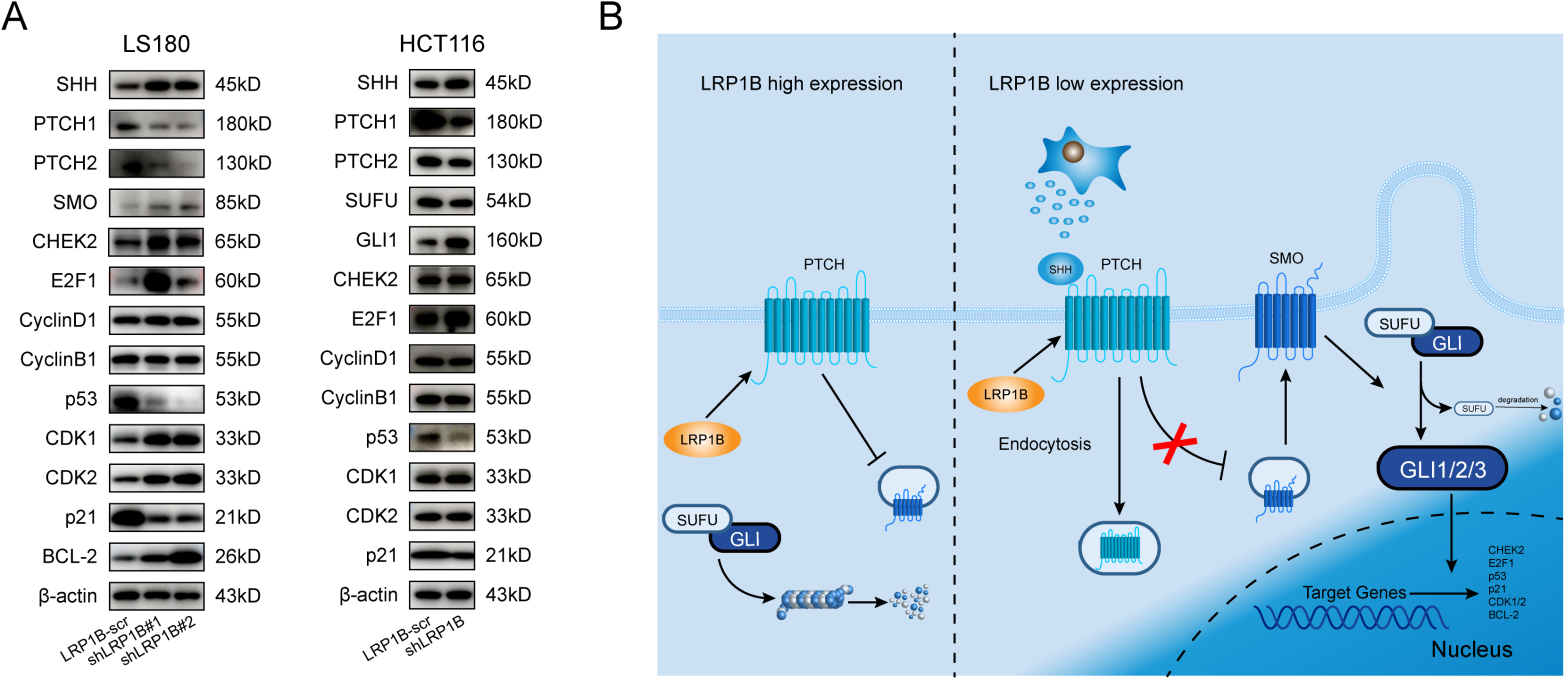
Association of LRP1B with the Hedgehog signaling pathway in CRC. **(A)** Western blot analysis of SHH, PTCH1, PTCH2, SMO, SUFU, GLI1, CHEK2, E2F1, Cyclin D1, Cyclin B1, p53, CDK1, CDK2, p21, BCL-2, and β-actin in LS180 and HCT116 cells with or without knockdown. **(B)** Potential mechanism of LRP1B in the suppression of CRC.

Next, according to Western blotting, knocking down LRP1B increased the expression of SHH, SMO, GLI1, CDK1, CDK2, CHEK2, E2F1, and BCL-2 while decreased the protein levels of PTCH1, PTCH2, SUFU, p53, and p21. These findings were consistent with the above enrichment analysis and experimental functional verification, further demonstrating the negative regulation of Hh signaling by LRP1B expression (**Figure 7A**). In the Hh signaling pathway, PTCH1 is regarded as the primary regulator (46, 47), and it modulates the intracellular localization of Cyclin B1, thereby linking its tumor-suppressive role to the regulation of cellular division (48). Therefore, we reasoned that the mechanism by which LRP1B inhibits CRC through the Hedgehog signaling pathway is shown in **Figure 7B**.

## 4 Discussion

The mutation of LRP1B has been identified in various cancers, including hepatocellular carcinoma (HCC) (7), ovarian cancer (OC) (49), glioblastoma (GB) (50), Merkel cell carcinoma (51), and gastric cancer (GC) (52), and is speculated to play a negative regulatory role in cancers. Simultaneously, LRP1B mutation has been reported to be critical in promoting immunotherapy in NSCLC patients (53, 54). Its mutation frequency has also been observed to increase after anti-EGFR therapy in CRC patients (55). Moreover, LRP1B variants have been associated with progression-free survival in patients receiving lenvatinib combined with immune checkpoint inhibitors following early hepatocellular carcinoma recurrence (56). However, due to its large size—comprising 4,599 amino acids encoded by a 13,800-base-pair mRNA—LRP1B is one of the largest transmembrane receptors (57). Although segmented amplification has been proposed to achieve overexpression of LRP1B (58), the varying infectivity of tumor cells and the specificity of antibodies continue to limit its validation. As a result, the underlying mechanism behind the role of LRP1B remains unclear.

The Hh signaling pathway is instrumental in regulating various aspects of animal development, including tissue homeostasis, regenerative mechanisms (59), and the maintenance of stem cells and tissues (14). However, aberrant activation of the Hh pathway has been linked to tumorigenesis, disease progression, metastasis, and drug resistance in multiple cancers, including basal cell carcinoma (BCC) (60), medulloblastoma (MB) (61), as well as various solid and hematological malignancies (14). Furthermore, research has implicated abnormal Hh signaling in the pathogenesis of breast (62), lung (48), pancreatic (63), and prostate cancers (64, 65), highlighting its potential role in the development of these diseases. Hh signaling pathways can be classified into canonical and noncanonical, both of which have been implicated in the pathogenesis of CRC (66). These pathways may influence CRC progression by interacting with other signaling pathways or regulating secretion mechanisms (67). Experimental studies have confirmed that phosphorylated c-Jun, activated by kinase JNK, prevents Gli2 from undergoing proteasomal-ubiquitin degradation through the PGE2-JNK signaling axis, thereby promoting Hh activation and colorectal cancer cell proliferation (68). Additionally, SRC-1, a member of the steroid receptor coactivator (SRC) family, has been identified as an enhancer of Gli2-mediated Hh signaling, contributing to CRC progression (69). The G-protein-coupled receptor 126 (GPR126) engendered increased transcription and translation of histone deacetylase 2 (HDAC2), which regulated Gli2 expression and enhanced colorectal cancer cell proliferation (70). CTCC-binding factor (CTCF) was verified to enhance malignant behaviors and chemotherapy resistance for 5-FU in CRC via the p53-Hedgehog axis (71). A series of analysis assays showed that the Hedgehog-Gli signaling pathway was necessary for increasing resistance to 5-fluorouracil in CRC cells (72). Ursolic acid (UA), a pentacyclic triterpenoid, may inhibit AKT signaling-dependent activation of the Smo-independent noncanonical Hedgehog pathway to protect against CRC (73). All of above highlighted the clinical utility of Hh signaling factors for CRC.

In the present study, we observed decreased LRP1B expression in CRC, and high LRP1B expression tended to be associated with better survival. The core gene in the PPI network SNAP25, associated with the microenvironment and immune response, has been identified as a predictor of poor outcomes in colon cancer (74). Additionally, we observed that the low-expression group exhibited a high mutational load, with FBXW7, SOX9, AMER1, SMAD4, ARID1A, and B2M being the most frequently mutated genes in colorectal cancer. Remarkably, in the low-expression group, LRP1B was found to be co-mutated with eight out of the top 25 significantly mutated genes. However, in the high-expression group, LRP1B was co-mutated with 18 major mutated genes. The co-occurrence of mutations in these genes was more pronounced in the low-expression group. These findings indicate a potential inverse relationship between LRP1B expression and mutation occurrence. From the single-cell analysis, we found that the expression of LRP1B was associated with immune cell infiltration, especially T cells.

Knocking down LRP1B promoted proliferation, accelerated the cell cycle, and inhibited apoptosis in colorectal cells. These results suggested that LRP1B was a tumor suppressor gene in CRC. Furthermore, we performed pathway enrichment analysis to analyze the possible mechanisms of LRP1B in CRC and found that the enrichment of Hedgehog pathway-related gene set signatures is notably related to LRP1B expression, besides cell cycle-related gene. Meanwhile, we proved that silencing LRP1B enhanced SHH, GLI1, CDK1, CDK2, CHEK2, E2F1, and BCL-2 expressions, while it decreased PTCH1, PTCH2, SUFU, p53, and p21 levels. Hence, we have reasonable speculation that LRP1B regulated cell cycle and apoptosis signals by influencing members of the Hh pathways. Several lines of evidence supported that m6A methylation of PTCH1 facilitated the hepatic stellate cell activation (75) and stem cell properties of esophageal cancer (76). PTCH2 ubiquitination has been proven to be involved in directing differentiation of embryonic stem cells (77). SUFU can be hydroxylated by the complex of P4HA2 and KIF7, which inhibits its function and amplifies Hh signaling in B-cell lymphoma (78). The sumoylation and phosphorylation of SMO have also been reported to be important in the Hh signaling pathway (79). All of these studies provided possible directions for potential target modifications of LRP1B in the Hh pathway.

In conclusion, our results demonstrate that high LRP1B expression is also associated with the infiltration of immune cells, such as dendritic cells, endothelial cells, and macrophages, in the immune microenvironment of CRC. The substantial disparity in drug response between the high- and low-expression groups highlights the potential of LRP1B as a valuable indicator for guiding drug therapy. Meanwhile, LRP1B may function as a tumor suppressor factor in CRC. Knockdown of LRP1B can activate the Hh pathway in tumor cells, inhibiting apoptosis and improving proliferation along with other malignant biological behaviors. Additionally, LRP1B is a promising target for CRC in immunotherapy or targeted therapy.

## Data Availability

The datasets presented in this study can be found in online repositories. The names of the repository/repositories and accession number(s) can be found in the article/**Supplementary Material**.
