# Supplementary material for: Molecular characteristics and cancer immunity of LRP1B and its relationship with the Hedgehog signaling pathway in colorectal cancer
Source: Front Immunol. 2025 Mar 18;16:1567102. doi: 10.3389/fimmu.2025.1567102 (PMC11959038; doi:10.3389/fimmu.2025.1567102)
Supplement: Supplementary file 1 [file DataSheet1.docx]

Supplementary Material

# Supplementary Figures and Tables

## Supplementary Figures

**
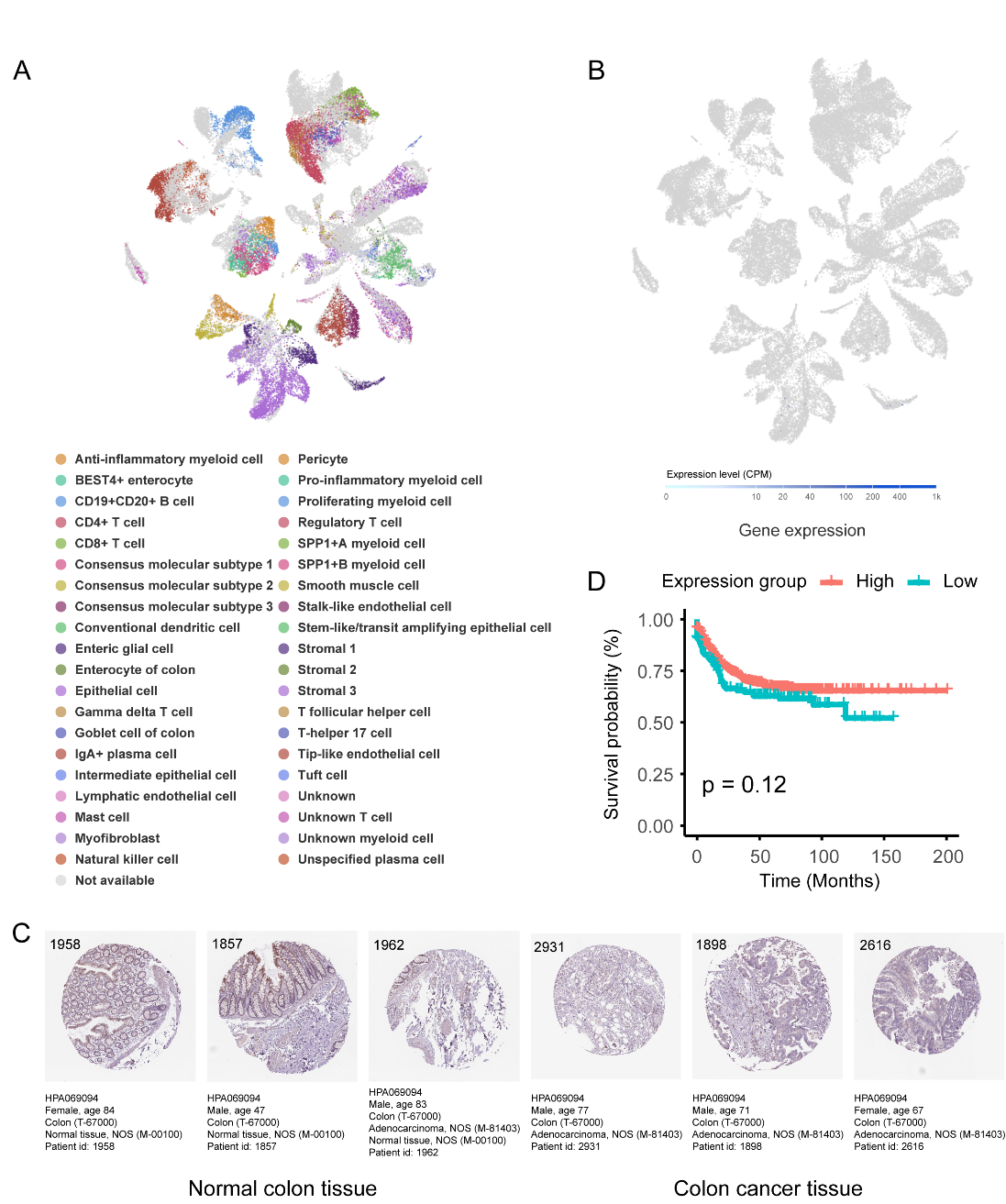
**

**Supplementary Figure 1.** UMAP plot showing the LRP1B expression site (A), and the annotation and color codes for reference cell types (B) in the CRC cell lines. (C) The IHC images of LRP1B in cancer and normal tissue from HPA platform. (D) Kaplan-Meier curves of progression-free survival (PFS) between high- and low-expression group.


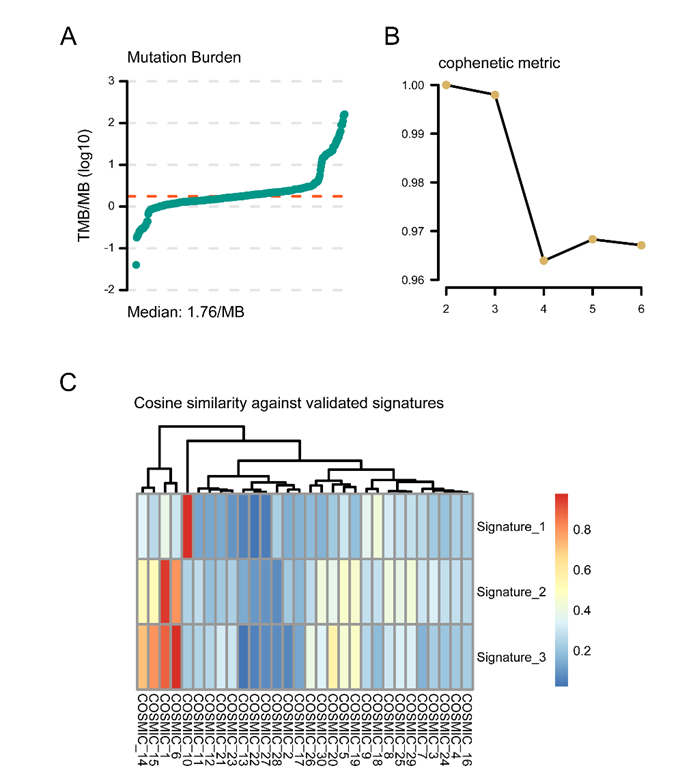


**Supplementary Figure 2.** (A) Dot plot showing the tumor mutation burdens across samples. (B) Line chart showing the exploration of cophenetic metric of extracted signatures. (C) Heatmap showing cosine similarity against validated signatures of extracted signatures.


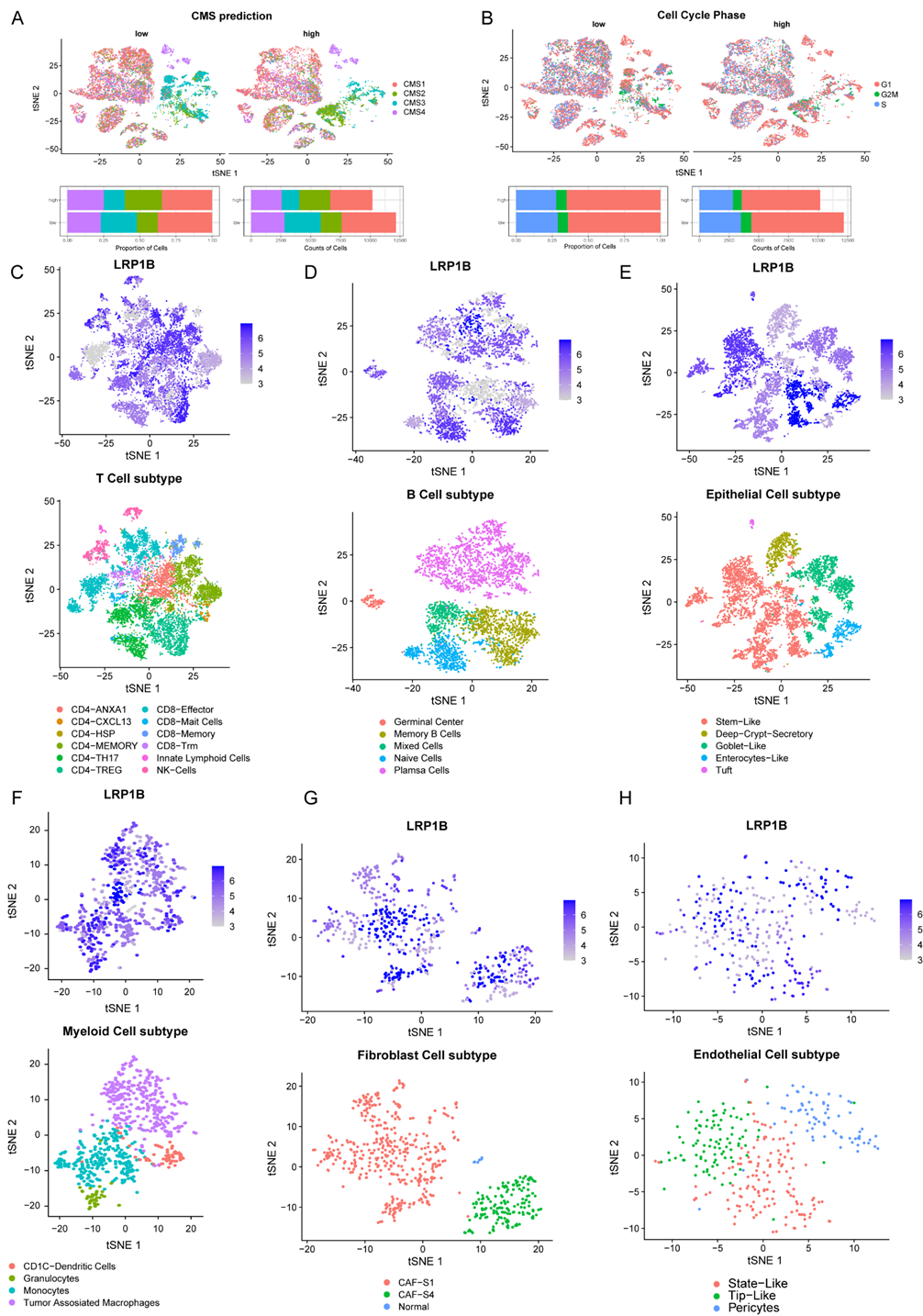


**Supplementary Figure 3**. tSNE plots (Upper) showed the CMS prediction (A) and the cell cycle phase (B) analysis in the high and low expression of LRP1B groups. Bar plots (Lower) represented the cell proportion and counts in the groups. tSNE plots revealed the groups and cell subtypes of T cells (C), B cells (D), epithelial cells (E), myeloid cells (F), fibroblast cells (G), and endothelial cells (H).


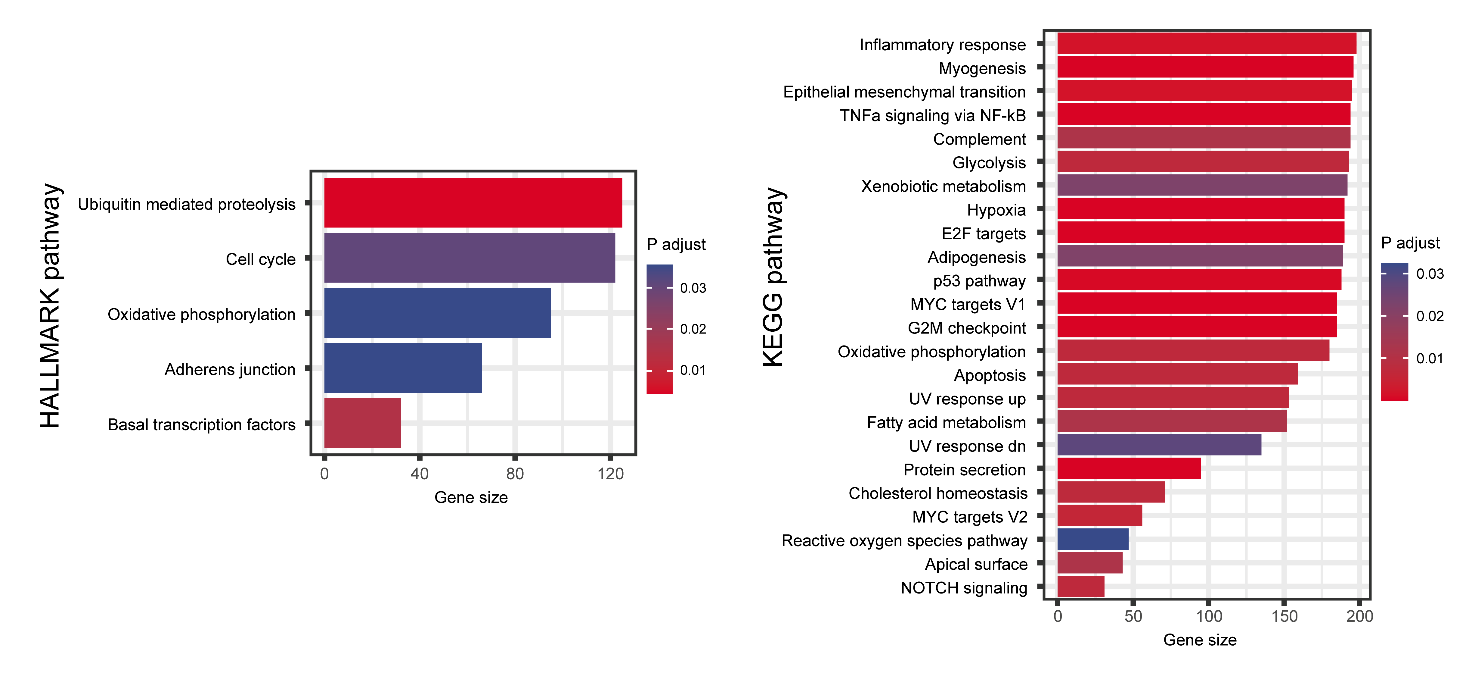


**Supplementary Figure 4**. Bar plot presenting the result of HALLMARK and KEGG biological pathway enrichment analysis between different expression groups in GSE39582.

## Supplementary Tables

| **Gene** | **HR** | **Z** | **P value** | **Lower** | **Upper** |
| --- | --- | --- | --- | --- | --- |
| TCEAL5 | 1.19717104 | 2.518248315 | 0.011794014 | 1.040703173 | 1.377163571 |
| NLGN1 | 1.213424062 | 3.508108554 | 0.000451305 | 1.08911854 | 1.351917079 |
| NTRK3 | 1.136293113 | 2.359704437 | 0.0182895 | 1.021880761 | 1.263515361 |
| GRIN2A | 1.124500235 | 1.994042375 | 0.046147424 | 1.002007343 | 1.261967578 |
| SUSD5 | 1.137388501 | 2.33672208 | 0.01945364 | 1.020973293 | 1.267077806 |
| ATCAY | 1.165152503 | 2.551819322 | 0.010716207 | 1.036087575 | 1.310294987 |
| ATRNL1 | 1.113133041 | 1.96242845 | 0.049712623 | 1.000134607 | 1.238898403 |
| ANK2 | 1.124131187 | 2.198067091 | 0.027944325 | 1.012755693 | 1.247754947 |
| C1orf95 | 1.08188165 | 1.981010769 | 0.047590066 | 1.000836498 | 1.169489629 |
| TCEAL2 | 1.11688132 | 2.706963252 | 0.006790177 | 1.030974124 | 1.209946839 |
| RBM20 | 1.154986389 | 2.310702257 | 0.020849307 | 1.022111925 | 1.305134522 |
| SALL2 | 1.151688119 | 2.206677239 | 0.027336617 | 1.015915128 | 1.305606627 |
| KIAA2022 | 1.144851695 | 2.335588886 | 0.019512678 | 1.021994224 | 1.28247829 |
| FILIP1 | 1.164371227 | 2.333619988 | 0.019615629 | 1.024666349 | 1.323123721 |
| MYH11 | 1.083347258 | 2.088086767 | 0.036790009 | 1.004924208 | 1.167890346 |
| RNF150 | 1.123765354 | 2.264167225 | 0.023563832 | 1.015800796 | 1.243204942 |
| ZNF676 | 1.219139882 | 2.178484431 | 0.02936999 | 1.020074519 | 1.457052426 |
| SYNPO2 | 1.095526544 | 2.011848917 | 0.044235866 | 1.002355694 | 1.197357801 |
| SGCG | 1.208892141 | 2.036171612 | 0.041733126 | 1.007125314 | 1.451080802 |
| SV2B | 1.105912954 | 2.148039078 | 0.031710651 | 1.008853394 | 1.2123104 |
| GPIHBP1 | 1.132859385 | 2.223794514 | 0.026162273 | 1.014909763 | 1.264516741 |
| SYNM | 1.091178745 | 2.073804328 | 0.038097487 | 1.004801499 | 1.184981367 |
| PDZD4 | 1.17496721 | 2.960071537 | 0.003075676 | 1.055988818 | 1.307350912 |
| ANGPTL7 | 1.117020012 | 1.976844538 | 0.048059206 | 1.000945426 | 1.246555181 |
| TACR1 | 1.124802356 | 2.244893182 | 0.024774991 | 1.015039074 | 1.24643511 |
| ZNF229 | 1.175975067 | 2.481395225 | 0.013086918 | 1.034649376 | 1.336604836 |
| KIF5A | 1.126709222 | 2.502072295 | 0.012346871 | 1.026185204 | 1.237080467 |
| BCHE | 1.126461814 | 2.828517489 | 0.004676415 | 1.037243183 | 1.223354599 |
| BHMT2 | 1.134788651 | 2.299483945 | 0.021477474 | 1.01884525 | 1.263926276 |
| SEMA3G | 1.165251744 | 2.294928729 | 0.021737209 | 1.022573515 | 1.327837663 |
| AGTR1 | 1.092773541 | 2.117900723 | 0.034183476 | 1.006637914 | 1.18627959 |
| MAP6 | 1.220774848 | 3.319300042 | 0.000902434 | 1.085124141 | 1.373383168 |
| DTNA | 1.175202438 | 3.615120055 | 0.000300208 | 1.076714534 | 1.282699105 |
| CNTN2 | 1.190154362 | 3.312428492 | 0.000924898 | 1.073665068 | 1.31928238 |
| KCNA5 | 1.171887327 | 2.057784639 | 0.039610811 | 1.007568586 | 1.363003894 |
| CILP | 1.080542179 | 2.215234699 | 0.026743959 | 1.008966335 | 1.157195596 |
| LINC02731 | 1.171908914 | 2.467913269 | 0.013590324 | 1.033189129 | 1.329253729 |
| DLG2 | 1.128717637 | 2.079776414 | 0.037546045 | 1.006999724 | 1.265147819 |
| MPPED2 | 1.173698516 | 2.564711203 | 0.010326171 | 1.038487131 | 1.326514471 |
| NPTX1 | 1.136042339 | 2.956084929 | 0.003115713 | 1.043918176 | 1.236296317 |
| LMOD1 | 1.1062369 | 2.060688958 | 0.039332724 | 1.004947248 | 1.217735639 |
| GALNT13 | 1.145465878 | 3.092089006 | 0.001987532 | 1.050982512 | 1.248443303 |
| NTNG1 | 1.161146812 | 2.528159258 | 0.011466233 | 1.034149115 | 1.30374034 |
| KCNK2 | 1.120283665 | 2.192977529 | 0.028308998 | 1.0121417 | 1.239980024 |
| TMEM90A | 1.188090885 | 2.329323203 | 0.01984195 | 1.027705927 | 1.373505703 |
| MYOM1 | 1.176456071 | 2.684273713 | 0.007268757 | 1.044825503 | 1.324669894 |
| GAP43 | 1.143571709 | 2.956099839 | 0.003115562 | 1.046244989 | 1.249952226 |
| CORO2B | 1.289352666 | 4.097922562 | 4.17E-05 | 1.141781171 | 1.455997295 |
| TNS1 | 1.184549032 | 2.97873426 | 0.002894417 | 1.059634784 | 1.324188703 |
| LEP | 1.110184248 | 2.310908077 | 0.020837933 | 1.016000408 | 1.213098986 |
| CLU | 1.150660922 | 2.712753069 | 0.006672681 | 1.039711616 | 1.273449807 |
| JPH4 | 1.203769349 | 2.672726831 | 0.007523749 | 1.050701334 | 1.379136581 |
| EPHA7 | 1.11289634 | 2.327581236 | 0.019934351 | 1.01703767 | 1.217789961 |
| SNAP25 | 1.101904529 | 2.053927232 | 0.039982735 | 1.004449267 | 1.20881525 |
| SCN4A | 1.206215265 | 3.173364183 | 0.001506834 | 1.074321909 | 1.354301029 |
| NPR1 | 1.154549634 | 2.141982998 | 0.03219485 | 1.012286927 | 1.316805366 |
| IGSF10 | 1.124991802 | 2.41300019 | 0.015821811 | 1.022358459 | 1.237928383 |
| LRRC4C | 1.133573995 | 2.419400244 | 0.015546124 | 1.024094078 | 1.254757772 |
| SLIT3 | 1.117546161 | 2.084382812 | 0.03712536 | 1.006655829 | 1.240651855 |
| ADCY5 | 1.108933929 | 2.285164429 | 0.022303188 | 1.014823465 | 1.211771803 |
| PCP4L1 | 1.118022391 | 2.209964042 | 0.027107658 | 1.012700248 | 1.234298174 |
| CCBE1 | 1.110457583 | 2.367131733 | 0.017926551 | 1.018185118 | 1.21109219 |
| AKAP12 | 1.126495219 | 2.423680816 | 0.015364103 | 1.023050916 | 1.240399141 |
| GPR133 | 1.134592815 | 2.435806707 | 0.014858622 | 1.024974774 | 1.255934183 |
| FXYD6 | 1.14042359 | 2.092556493 | 0.036388764 | 1.008360757 | 1.289782408 |
| ODZ1 | 1.134268394 | 2.332979494 | 0.019649222 | 1.02034821 | 1.260907579 |
| PTPRZ1 | 1.137213565 | 2.955969215 | 0.003116882 | 1.044277234 | 1.238420842 |
| CRTAC1 | 1.148423298 | 3.249443511 | 0.00115631 | 1.056453356 | 1.248399716 |
| EML1 | 1.153203266 | 2.081354528 | 0.037401468 | 1.008348199 | 1.318867603 |
| SLC18A3 | 1.131090197 | 2.114534008 | 0.034469683 | 1.009045122 | 1.267896754 |
| CADM3 | 1.086232066 | 2.131954259 | 0.033010607 | 1.006695137 | 1.172053045 |
| POPDC2 | 1.185628828 | 2.759251618 | 0.005793391 | 1.050560681 | 1.338062374 |
| CHRDL1 | 1.072603827 | 2.043457886 | 0.041007132 | 1.002867887 | 1.147188962 |
| SPARCL1 | 1.13225761 | 1.981160551 | 0.047573272 | 1.001329852 | 1.28030468 |
| ADAMTS1 | 1.190117426 | 2.536307978 | 0.011202816 | 1.040343677 | 1.36145345 |
| SNAP91 | 1.14991959 | 2.377491922 | 0.017430825 | 1.024835682 | 1.29027032 |
| HSPB7 | 1.123870401 | 2.826382789 | 0.004707697 | 1.036446532 | 1.21866844 |
| C7 | 1.095946149 | 2.486941714 | 0.012884651 | 1.019603344 | 1.178005122 |
| HS6ST3 | 1.278971899 | 4.185371413 | 2.85E-05 | 1.139775075 | 1.435168354 |
| NGFR | 1.136053294 | 2.852513373 | 0.004337499 | 1.040720753 | 1.240118525 |
| PTH1R | 1.244234167 | 3.666925107 | 0.000245485 | 1.107075225 | 1.39838615 |
| TNXB | 1.122590303 | 2.344220325 | 0.019066904 | 1.019135891 | 1.236546568 |
| RBPMS2 | 1.151251608 | 2.692523255 | 0.007091359 | 1.03906494 | 1.275550942 |
| AOC3 | 1.151852148 | 2.565652355 | 0.010298198 | 1.033937488 | 1.2832143 |
| CRYAB | 1.176331559 | 2.792848644 | 0.005224613 | 1.049623185 | 1.31833591 |
| PDZRN4 | 1.125421013 | 2.751901334 | 0.005925037 | 1.034587785 | 1.224229084 |
| JPH2 | 1.15428772 | 2.892709407 | 0.003819345 | 1.04735277 | 1.272140753 |
| C15orf59 | 1.143229933 | 2.193373708 | 0.028280465 | 1.0143465 | 1.288489367 |
| NUDT10 | 1.153631882 | 2.426847623 | 0.015230651 | 1.027875865 | 1.294773586 |
| MGP | 1.109645691 | 1.991727057 | 0.046401013 | 1.001660568 | 1.22927227 |
| KIF1A | 1.103118859 | 2.485036361 | 0.01295382 | 1.020953172 | 1.191897189 |
| FABP4 | 1.093304959 | 2.743227014 | 0.006083861 | 1.025797554 | 1.165255004 |
| ATP1B2 | 1.176950867 | 2.346186138 | 0.018966633 | 1.027183478 | 1.348554929 |
| INA | 1.123978868 | 2.374003811 | 0.017596369 | 1.020592825 | 1.237837916 |
| PYGM | 1.137564119 | 2.454437742 | 0.01411051 | 1.026306227 | 1.260883049 |
| CPEB1 | 1.133981074 | 2.0294494 | 0.042412538 | 1.004314248 | 1.280389161 |
| CNGA3 | 1.133479935 | 2.049150216 | 0.040447428 | 1.005468066 | 1.277789724 |
| DPYSL5 | 1.184835813 | 2.757959364 | 0.005816343 | 1.050297795 | 1.336607494 |
| PRELP | 1.119043182 | 2.696339464 | 0.007010618 | 1.031193501 | 1.214376974 |
| C20orf194 | 1.162956025 | 2.249286391 | 0.024494281 | 1.019608178 | 1.326457305 |
| MYL9 | 1.148821432 | 2.242199683 | 0.02494847 | 1.01761677 | 1.296942742 |
| PLIN4 | 1.087601556 | 2.504672071 | 0.012256497 | 1.018430366 | 1.161470812 |
| SLC6A15 | 1.161836559 | 3.147179847 | 0.001648535 | 1.058217089 | 1.275602335 |
| SEMA3D | 1.091747798 | 2.028657266 | 0.042493211 | 1.002976777 | 1.188375726 |
| NBEA | 1.166343937 | 3.158565305 | 0.001585478 | 1.060130034 | 1.28319936 |
| TUBB2B | 1.145040411 | 3.298844156 | 0.000970838 | 1.056509039 | 1.240990371 |
| DACT3 | 1.167719108 | 2.506507823 | 0.012193036 | 1.034387182 | 1.318237446 |
| PACSIN1 | 1.146820935 | 2.113193368 | 0.034584221 | 1.009983027 | 1.302198374 |
| SGCA | 1.09951868 | 2.216593658 | 0.026650871 | 1.011044564 | 1.195734957 |
| HMP19 | 1.130741494 | 2.567087859 | 0.010255662 | 1.029486333 | 1.241955609 |
| NACAD | 1.202701298 | 2.856953929 | 0.004277279 | 1.059660918 | 1.365050261 |
| MAP2 | 1.20191097 | 2.98890804 | 0.002799764 | 1.065359964 | 1.355964209 |
| THBS4 | 1.061254937 | 2.16301006 | 0.030540406 | 1.005596491 | 1.119994005 |
| NBLA00301 | 1.096243851 | 2.034122842 | 0.041939211 | 1.003355677 | 1.197731381 |
| SOX10 | 1.116704162 | 2.488532439 | 0.012827153 | 1.023722244 | 1.218131375 |
| GEFT | 1.194111613 | 2.736484197 | 0.006209958 | 1.051629359 | 1.355898379 |
| TLL1 | 1.122324681 | 2.105707213 | 0.035229793 | 1.008019363 | 1.24959176 |
| CHRNA3 | 1.094730977 | 1.988945144 | 0.046707258 | 1.001319682 | 1.196856442 |
| CHRNB4 | 1.139579913 | 1.997381677 | 0.045783737 | 1.002450695 | 1.295467583 |
| TAGLN3 | 1.128868208 | 2.304718337 | 0.021182353 | 1.018297574 | 1.251445023 |
| C12orf53 | 1.234080203 | 3.143533287 | 0.001669214 | 1.082409581 | 1.407003387 |
| PALM | 1.142981475 | 2.254280027 | 0.024178557 | 1.017601002 | 1.283810304 |
| GRIK5 | 1.134410611 | 2.38848479 | 0.016918009 | 1.022884032 | 1.2580971 |
| WBSCR17 | 1.169983296 | 2.635146749 | 0.008410091 | 1.041044117 | 1.314892318 |
| GPM6A | 1.081518901 | 2.122509039 | 0.033795014 | 1.006019469 | 1.162684391 |
| NTN1 | 1.163226991 | 2.376216762 | 0.017491184 | 1.026839925 | 1.317729279 |
| SCN3B | 1.114061689 | 1.970590885 | 0.04877069 | 1.000582654 | 1.240410717 |
| CNN1 | 1.112479509 | 2.5251739 | 0.011564105 | 1.024145232 | 1.208432768 |
| DYNC1I1 | 1.124274615 | 2.57090269 | 0.010143382 | 1.028227248 | 1.229293829 |
| TRIM9 | 1.124162736 | 2.357725767 | 0.018387271 | 1.01994129 | 1.239033923 |
| CLDN11 | 1.226142545 | 3.763734703 | 0.000167394 | 1.102638815 | 1.363479609 |
| PTPRN | 1.172214364 | 3.729586013 | 0.000191795 | 1.078307458 | 1.27429937 |
| SPEG | 1.126893661 | 2.899192352 | 0.003741253 | 1.03946077 | 1.221680856 |
| PSD | 1.097079658 | 2.082734206 | 0.037275458 | 1.005476455 | 1.197028304 |
| CCDC78 | 1.154032617 | 2.594345317 | 0.009477125 | 1.035652014 | 1.28594476 |
| EEF1A2 | 1.106011944 | 2.524208035 | 0.011595928 | 1.022778918 | 1.196018415 |
| LYNX1 | 1.122013536 | 2.01333108 | 0.044079811 | 1.00305626 | 1.255078527 |
| UCHL1 | 1.129849864 | 2.796614425 | 0.005164112 | 1.037198723 | 1.23077737 |
| SCG2 | 1.178863899 | 3.312692483 | 0.000924025 | 1.069502944 | 1.29940745 |
| NNAT | 1.141907967 | 2.391905387 | 0.016761162 | 1.024253113 | 1.273077706 |
| CRLF1 | 1.193038241 | 3.289548384 | 0.001003483 | 1.073946147 | 1.325336702 |
| HRASLS5 | 1.102232779 | 2.207047229 | 0.02731076 | 1.010956761 | 1.201749814 |
| WISP2 | 1.087093113 | 2.138527836 | 0.032473927 | 1.006997097 | 1.173559924 |
| STXBP5L | 1.150661654 | 2.659596498 | 0.007823432 | 1.037606933 | 1.2760345 |
| TACR2 | 1.133817069 | 2.740513886 | 0.006134319 | 1.036417816 | 1.240369596 |
| PCDH9 | 1.183958385 | 2.918643521 | 0.00351558 | 1.05703322 | 1.326124319 |
| SPTBN4 | 1.19776869 | 3.155749289 | 0.001600864 | 1.070772721 | 1.339826656 |
| DES | 1.062263966 | 2.058486345 | 0.039543471 | 1.002895138 | 1.125147277 |
| MAB21L2 | 1.105249586 | 2.029986677 | 0.042357894 | 1.003457836 | 1.21736719 |
| PNCK | 1.106050116 | 2.533416226 | 0.011295674 | 1.023077798 | 1.195751546 |
| PRPH | 1.113023427 | 2.534555666 | 0.011259004 | 1.024572444 | 1.209110353 |
| DCHS2 | 1.138569828 | 3.443232973 | 0.000574804 | 1.057495446 | 1.225859892 |
| GFRA3 | 1.11109818 | 2.073315444 | 0.038142932 | 1.005776209 | 1.227449162 |

**Supplementary Table S1.** Prognostic analysis of expression intergroup differential genes and cancer and paracancer differential genes.

| **Rank** | **Gene** | **Betweenness** |
| --- | --- | --- |
| 1 | SNAP25 | Betweenness: 2948.8718 |
| 2 | AGTR1 | Betweenness: 1564.2798 |
| 3 | MAP2 | Betweenness: 1421.2874 |
| 4 | CLU | Betweenness: 1417.9457 |
| 5 | CNTN2 | Betweenness: 1038.9172 |
| 6 | SNAP91 | Betweenness: 1029.3339 |
| 7 | SPARCL1 | Betweenness: 1015.6826 |
| 8 | DES | Betweenness: 988.99896 |
| 9 | GAP43 | Betweenness: 922.7456 |
| 10 | JPH2 | Betweenness: 922.6356 |
| 11 | CRYAB | Betweenness: 890.2234 |
| 12 | NLGN1 | Betweenness: 885.6518 |
| 13 | ANK2 | Betweenness: 856.1746 |
| 14 | MYH11 | Betweenness: 754.86505 |
| 15 | UCHL1 | Betweenness: 668.5944 |
| 16 | CILP | Betweenness: 646.40674 |
| 17 | ARHGEF25 | Betweenness: 641.10175 |
| 18 | DLG2 | Betweenness: 637.5938 |
| 19 | DTNA | Betweenness: 624.0039 |
| 20 | PTH1R | Betweenness: 612.0 |
| 21 | NTN1 | Betweenness: 569.34186 |
| 22 | BCHE | Betweenness: 561.0415 |
| 23 | SYNPO2 | Betweenness: 468.24286 |
| 24 | MYL9 | Betweenness: 438.52774 |
| 25 | NNAT | Betweenness: 412.0 |
| 26 | LEP | Betweenness: 412.0 |
| 27 | SLC18A3 | Betweenness: 378.96237 |
| 28 | MGP | Betweenness: 371.80865 |
| 29 | SPTBN4 | Betweenness: 370.3932 |
| 30 | PRELP | Betweenness: 366.92334 |
| 31 | SOX10 | Betweenness: 366.20703 |
| 32 | CNN1 | Betweenness: 326.73486 |
| 33 | LYNX1 | Betweenness: 318.68658 |
| 34 | INA | Betweenness: 308.94238 |
| 35 | SLIT3 | Betweenness: 296.7685 |
| 36 | GRIN2A | Betweenness: 285.5821 |
| 37 | NGFR | Betweenness: 256.51443 |
| 38 | PTPRZ1 | Betweenness: 223.21152 |
| 39 | KCNK2 | Betweenness: 220.29948 |
| 40 | STXBP5L | Betweenness: 218.8294 |
| 41 | GPM6A | Betweenness: 209.16374 |
| 42 | SCN4A | Betweenness: 208.66754 |
| 43 | RNF150 | Betweenness: 208.0 |
| 44 | ATP1B2 | Betweenness: 208.0 |
| 45 | NTNG1 | Betweenness: 189.72493 |
| 46 | POPDC2 | Betweenness: 177.61407 |
| 47 | THBS4 | Betweenness: 171.72302 |
| 48 | TAGLN3 | Betweenness: 164.8277 |
| 49 | TACR1 | Betweenness: 152.38922 |
| 50 | KIF5A | Betweenness: 151.63264 |
| 51 | CHRNB4 | Betweenness: 136.61429 |
| 52 | NTRK3 | Betweenness: 128.70738 |
| 53 | KIF1A | Betweenness: 124.93608 |
| 54 | SGCA | Betweenness: 123.48426 |
| 55 | TUBB2B | Betweenness: 91.84827 |
| 56 | SYNM | Betweenness: 86.88781 |
| 57 | RBM20 | Betweenness: 82.004074 |
| 58 | SCN3B | Betweenness: 79.82896 |
| 59 | GRIK5 | Betweenness: 52.44882 |
| 60 | LRRC4C | Betweenness: 41.527046 |
| 61 | PTPRN | Betweenness: 33.332676 |
| 62 | CADM3 | Betweenness: 30.202381 |
| 63 | PCDH9 | Betweenness: 26.325275 |
| 64 | KCNA5 | Betweenness: 25.857143 |
| 65 | TRIM9 | Betweenness: 23.507576 |
| 66 | CLDN11 | Betweenness: 21.16154 |
| 67 | DYNC1I1 | Betweenness: 20.184813 |
| 68 | LMOD1 | Betweenness: 9.066667 |

**Supplementary Table S2.** Genes in PPI network.
